# Supplementary material for: Self-regulating arousal via pupil-based biofeedback
Source: Nat Hum Behav. 2023 Oct 30;8(1):43–62. doi: 10.1038/s41562-023-01729-z (PMC10810759; doi:10.1038/s41562-023-01729-z)
Supplement: Supplementary file 2 — Reporting Summary [file 41562_2023_1729_MOESM2_ESM.pdf]

nature portfolio

Corresponding author(s): Sarah Nadine Meissner, Nicole Wenderoth

Last updated by author(s): Sep 11, 2023

Reporting Summary

Nature Portfolio wishes to improve the reproducibility of the work that we publish. This form provides structure for consistency and transparency in reporting. For further information on Nature Portfolio policies, see our Editorial Policies and the Editorial Policy Checklist.

Statistics

For all statistical analyses, confirm that the following items are present in the figure legend, table legend, main text, or Methods section.

n/a	Confirmed	
☐	☒	The exact sample size (n) for each experimental group/condition, given as a discrete number and unit of measurement
☐	☒	A statement on whether measurements were taken from distinct samples or whether the same sample was measured repeatedly
☐	☒	The statistical test(s) used AND whether they are one- or two-sided<br>*Only common tests should be described solely by name; describe more complex techniques in the Methods section.*
☒	☐	A description of all covariates tested
☐	☒	A description of any assumptions or corrections, such as tests of normality and adjustment for multiple comparisons
☐	☒	A full description of the statistical parameters including central tendency (e.g. means) or other basic estimates (e.g. regression coefficient) AND variation (e.g. standard deviation) or associated estimates of uncertainty (e.g. confidence intervals)
☐	☒	For null hypothesis testing, the test statistic (e.g. F, t, r) with confidence intervals, effect sizes, degrees of freedom and P value noted<br>*Give P values as exact values whenever suitable.*
☐	☒	For Bayesian analysis, information on the choice of priors and Markov chain Monte Carlo settings
☒	☐	For hierarchical and complex designs, identification of the appropriate level for tests and full reporting of outcomes
☐	☒	Estimates of effect sizes (e.g. Cohen's d, Pearson's r), indicating how they were calculated

Our web collection on statistics for biologists contains articles on many of the points above.

Software and code

Policy information about availability of computer code

Data collection	For the collection of pupil data during Experiments 1A, 1B and 3, we used MATLAB (version 2013a) and the Tobii_TX300 SDK for MATLAB version 3; for response recording of behavioral data, we used the MATLAB-based presentation software Psychtoolbox 3.0.17. For the collection of (f)MRI, pulse and respiratory data, we used the Philips software version 5.4.1 (Philips 3T Ingenia; Philips; Philips Medical Systems; Invivo); for recording of pupil data during fMRI (Experiment 2), we used Matlab (2019a) and the Eyelink 1000 plus software of SR Research; for the recording of cardiovascular and respiratory data (in Experiment 1B), we used the Biopac MP 160 system with the accompanying AcqKnowledge software version 5.0
Data analysis	For (pre-)processing of pupil data, open-source software from Kret & Sjak-Shie (2018) and Matlab code (2018a) (e.g., for baseline correction) was used. For (pre-)processing of cardiovascular data, we used the Matlab-based toolbox physiozoo (Matlab version 2021a) and Matlab code (Matlab version 2018a). For (f)MRI data analyses, we used FSL version 6.0.5.2 (including the implemented analyses tools BET, MCFLIRT, FEAT, FNIRT, FLIRT, FILM, FLOBS, MELODIC, and PNM), FreeSurfer version 6.0. For all statistical analyses, we used IBM SPSS version 28; R version 4.1.2/4.2.2 including the packages WRS2 (version 1.1-3) and rmcorr (version 0.4.5, 0.6.0), JASP version 0.16.2, and the Matlab-based SPM-1D toolbox (M.0.4.8).

For manuscripts utilizing custom algorithms or software that are central to the research but not yet described in published literature, software must be made available to editors and reviewers. We strongly encourage code deposition in a community repository (e.g. GitHub). See the Nature Portfolio guidelines for submitting code & software for further information.

Data

Policy information about availability of data

All manuscripts must include a data availability statement. This statement should provide the following information, where applicable:

- Accession codes, unique identifiers, or web links for publicly available datasets
- A description of any restrictions on data availability
- For clinical datasets or third party data, please ensure that the statement adheres to our policy

Processed data are openly available on the ETH Library Research Collection: https://doi.org/10.3929/ethz-b-000630621
For fMRI regions of interest analyses (ROI), we used probabilistic anatomical/cytoarchitectonic atlases to define the locations of the ROIs (Brainstem Navigator; https://www.nitrc.org/projects/brainstemnavig/; https://www.fz-juelich.de/en/inm/inm-7/resources/jubrain-anatomy-toolbox) as well as the complete brainstem (control analyses; https://www.fil.ion.ucl.ac.uk/spm/toolbox/TPM/).

Human research participants

Policy information about studies involving human research participants and Sex and Gender in Research.

Reporting on sex and gender	The reported results apply to the female and male sex: our sample included male and female participants. This information was determined based on self-reports of the participants.<br>We did not conduct sex-/gender-based analyses in addition to our group level analyses since our study included both sexes and was not powered to conduct separate analyses
Population characteristics	See behavioral and social sciences study design information
Recruitment	Healthy participants of the present study were recruited via online advertisement on University web pages. We are not aware of any self-selection bias, however, one bias that may impact the results is that the majority of the participants were young healthy university students in Switzerland. All participants gave written informed consent.
Ethics oversight	All experimental protocols were approved by the research ethics committee of the canton of Zurich (KEK-ZH 2018-01078)

Note that full information on the approval of the study protocol must also be provided in the manuscript.

Field-specific reporting

Please select the one below that is the best fit for your research. If you are not sure, read the appropriate sections before making your selection.

☐ Life sciences ☒ Behavioural & social sciences ☐ Ecological, evolutionary & environmental sciences

For a reference copy of the document with all sections, see nature.com/documents/nr-reporting-summary-flat.pdf

Behavioural & social sciences study design

All studies must disclose on these points even when the disclosure is negative.

Study description	The present study is a quantitative experimental study
Research sample	Participants were mainly healthy university students (undergraduate, graduate students) from different Universities in Zürich/ Switzerland. Since it's mainly including young adultss the sample is not representative considering the general population<br>In the pupil-NF group of Experiment 1A, participants were 24 +/- 5 years old (mean +/- SD), 16 female and 12 male; in control group I, participants had a mean age (+/- SD) of 24 +/- 5 years, 13 were female and 15 male. In control group II, participants were were 25 +/- 7 years old (mean +/- SD), 13 were female and 3 were male.<br>For Experiment 1B, participants had a mean age (+/- SD) of 26 +/- 7 years, 18 were female and 8 male. Experiments 2 and 3 included a subset of the participants of Experiments 1A and 1B.<br>Since the present study is to our knowledge the first proof-of-concept study of pupil-based biofeedback combined with (brainstem and whole-brain) fMRI consisting of multiple (training) sessions, we decided to recruit an accessible sample of mainly university students.
Sampling strategy	Participants were randomly assigned to a pupil-BF and control group I in Experiment 1A. Control group II was recruited after acquiring data of the pupil-BF and control group I.<br>For our pupil-based biofeedback paradigm, we did a power calculation (with a power level of 80%) based on pilot data of a single pupil-based BF session. Our final sample size of Experiment 1A including 28 participants for each group (Biofeedback and Control group I) furthermore exceeds the sample size of many other biofeedback studies. Since we were the first ones to combine pupil-based biofeedback with fMRI and an oddball task, we based our sample size estimations for Experiment 2 and 3 on previous fMRI studies showing a link between pupil size and brain (LC) activity and the oddball task, respectively.

Data collection	We used eye trackers (Tobii TX300 in Experiments 1 and 3; Eyelink 1000 Plus Experiment 2) for the collection of pupil data, an MRI scanner (Philips Ingenia, 3T) for fMRI data collection; ECG, a peripheral pulse sensor and a respiratory belt (Biopac MP 160 system for Experiment 1A and B; Invivo Philips Medical Systems for Experiment 2) to collect heart rate and respiratory data. Behavioral data was acquired and stored offline on a PC. Demographic data and questionnaire data (e.g., for exclusion criteria) were acquired using pen and pencil. During data acquisition, no-one except for researchers and participants were present. Furthermore, during all experiments, participants were either in a Faraday cage or MRI scanner with the experimenter being in the control room, thus participants could focus on themselves without the feeling of someone watching/influencing them during the experiments. Participants were blind regarding the experimental group and the hypotheses of the study; the researchers analyzing the data were not blind to the experimental groups during data collection but blind to the experimental condition/groups during preprocessing of all data.
Timing	Data for experiment 1A was collected from October 2019 to February 2020 (pupil-BF and control group I) and from April to June 2023 (control group II); Data for experiment 1B, 2 and 3 was collected between July 2020 and October 2021. This phase was prolonged due to the COVID-19 pandemic and respective regulations.
Data exclusions	In experiment 1A, pupil data of one participant had to be excluded due to too many missing data points (more than 30% missing data during baseline/self-regulation of pupil size for more than 50% of trials, pre-set exclusion criterion). Further ECG data (control group II) of 1 participant on day 2 and 1 participant for all days was corrupted after data saving. For 3 participants, there was incomplete trigger information due to technical issues with the trigger box for no feedback trials at day 3.<br>In experiment 1B, pupil data of one participant had to be excluded due to technical issues with the eye tracker and its recording. Further, ECG data of one session of a participant (experiment 1B) needed to be excluded due to poor data quality (non-detectable R-peaks)<br>In experiment 2, pulse rate data of 2 participants needed to be excluded due to technical issues during recording (whole brain session; n=1), and poor and noisy data quality with barely detectable R-peaks (brainstem session; n=1); brainstem fMRI data of 3 participants needed to be excluded due to excessive motion (pre-set criterion, n=1), distortions (n=1), and falling asleep inside the scanner (n=1); whole-brain fMRI data of 1 participant needed to be excluded due to distortions in frontal and temporal regions. Additionally, 1 run for 3 participants and 2 runs for 1 participant needed to be excluded due to excessive motion (pre-set criterion of half a voxel size mean displacement).<br>In experiment 3, pupil data of 2 participants needed to be excluded due to the same reasons as described for Experiment 1A (pre-set criterion). For 1 of these 2 participants, behavioral data was excluded as well (correct responses during the task deviated more than 3SD from the group mean)
Non-participation	No participant has declined participation after recruitment. One participant of the control group of Experiment 1A dropped out due to personal reasons after the first day of training; from Experiment 2 to 3, 3 participants dropped out due to personal reasons (e.g., moving, no time to participate)
Randomization	Participants were allocated randomly.

Reporting for specific materials, systems and methods

We require information from authors about some types of materials, experimental systems and methods used in many studies. Here, indicate whether each material, system or method listed is relevant to your study. If you are not sure if a list item applies to your research, read the appropriate section before selecting a response.

Materials & experimental systems

n/a	Involved in the study	
☒	☐	Antibodies
☒	☐	Eukaryotic cell lines
☒	☐	Palaeontology and archaeology
☒	☐	Animals and other organisms
☒	☐	Clinical data
☒	☐	Dual use research of concern

Methods

n/a	Involved in the study	
☒	☐	ChIP-seq
☒	☐	Flow cytometry
☐	☒	MRI-based neuroimaging

Magnetic resonance imaging

Experimental design

Design type	task-based fMRI with a block design
Design specifications	per fMRI session: 8 blocks à 4 trials of each condition (up- vs. downregulation). Each block was of ~2.5 minutes length and between each block, there was a 10 s break. Between the trials of a block there was a jittered break of 6-9 s. 1 fMRI run consisted of 2 blocks of each condition (~10 min) after which there was a break for the participants.
Behavioral performance measures	We did not record any behavioral data such as button presses but pupil size throughout the experiment. Participants were asked to apply pupil self-regulation while we simultaneously recorded fMRI data. On our control screen, we saw the pupil-based biofeedback as an immediate check of task performance (similar to the participants themselves) and

additionally investigated the pupil up- and downregulation (mean across all trials of each condition for each participant) and the standard deviation/standard error of the mean across participants in an offline anaylsis

Acquisition

Imaging type(s)	functional, structural
Field strength	3T
Sequence & imaging parameters	T1-weighted anatomical sequence: MPRAGE; 160 sagittal slices, TR/TE: 9.3/4.4 ms; voxel size: 0.7 mm^3; matrix size: 240 x 240, flip angle: 8°; field of view 240mm x 240 mm x 160 mm<br>TSE structural scan (not reported in the present analysis): 20 slices, 1.5 mm slice thickness, TR/TE: 500/10 ms; flip angle: 90°, in-plane resolution: 0.7 X 0.8 mm<br>brainstem fMRI: EPI, 39 sagittal slices (thickness 1.8mm); TR: 2.5s, TE: 26ms; flip angle 82°; SENSE acceleration factor: 2.1; in-plane resolution of 1.8 x 1.8 mm (+ 1 whole-brain single volume EPI image with the same parameters to improve co-registration). FOV: 210 mm x 187.6 mm x 70.2 mm<br>whole-brain fMRI: EPI, 40 slices (thickness: 2.7 mm); TR: 2.5s, TE: 30ms; flip angle: 85°, FOV: 223 mm x 223 mm x 116 mm, SENSE factor 2. In-plane resolution of 2.7 x 2.7 mm. (first 7 participants, EPI with slightly different sequence parameters: 36 slices with 3 mm thickness, FOV: 210 mm x 210 mm x 108 mm, SENSE factor 2.2; in plane resolution of 2 x 2 mm.; flip angle 82°, same TR/TE)
Area of acquisition	both whole-brain scanning and limited field of views were used (for brainstem imaging). The field of view was determined by our main regions of interested in the brainstem that needed to be covered (mainly Locus Coeruleus but also more anterior nuclei including the Nucleus Basalis of Meynert). The fourth ventricle aided orientation of the scans.

Diffusion MRI ☐ Used ☒ Not used

Preprocessing

Preprocessing software	We used FSL version 6.0.5.2 and FreeSurfer version 6.0 for preprocessing of fMRI data. Brain extraction was performed using FSL's automated brain extraction tool (BET; v2.1), motion correction using the Linear Image Registration Tool (MCFLIRT), spatial smoothing using a 3mm full width-at-half-maximum (FWHM) Gaussian kernel for the brainstem data and 5mm FWHM Gaussian kernel for the whole-brain data, and a 90s high-pass temporal filter as implemented via FSL's Expert Analysis Tool (FEAT, v6.0). For noise and artifact removal, see section below. For additional control analyses of the brainstem fMRI data, we re-analyzed the data without applying any spatial smoothing.
Normalization	brainstem fMRI data: Image co-registration from functional to MNI standard space was performed by (i) aligning the functional images of each run to each subject's whole-brain EPI image, using FSL's Linear Image Registration Tool (FLIRT) employing a mutual information cost function and six degrees of freedom; (ii) registering whole-brain EPIs to structural T1-weighted images, using a mutual information cost function six and degrees of freedom, and then optimised using boundary-based registration (BBR); (iii) co-registering structural images to the MNI152 template via FSL's nonlinear registration tool (FNIRT) using 12 degrees of freedom, and applying the resulting warp fields to the functional images. Each co-registration step was visually inspected using Freeview (FreeSurfer, version 6.0).<br>Whole-brain data: similar to the description above: Functional EPI-images were aligned to structural T1-weighted images using linear registration (FLIRT). Structural images were aligned to the MNI standard space using nonlinear registration (FNIRT)
Normalization template	we used the MNI152 standard-space structural template image provided by FSL
Noise and artifact removal	we implemented motion correction (using the Linear Image Registration tool implemented in FSL; MCFLIRT) for both whole-brain and brainstem data, independent component analyses (using MELODIC implemented in FSL) and physiological noise modeling (using FSL's PNM, an extended version of RETROICOR; the latter only for brainstem data). For PNM, we used physiological recordings from the peripheral pulse sensor and respiration data during scanning
Volume censoring	we did not apply additional volume censoring in addition to the noise and artifact removal described above.

Statistical modeling & inference

Model type and settings	To assess fMRI task-related activity, we used univariate models for data analyses. For the brainstem session's first-level analyses, we implemented fixed-effects analyses using FEAT with FMRIB's Improved Linear Model (FILM) integrated for local autocorrelation. To account for potential differences in the brainstem's HRF, we used the FLOBS toolkit implemented in FSL with the default optimal basis function to model the HRF. PNM voxelwise confound lists and ICA noise component time series were added as nuisance regressors. For second level and third level analyses we, however, only passed up the canonical HRF parameter estimates. Third level analyses were performed using mixed effects analyses.<br>For the whole-brain session's data analyses, we took a similar approach, however, we used a double gamma HRF (instead of the optimal basis functions to model the HRF) and its first temporal derivative. White matter, cerebrospinal fluid time series, motion parameters were added as nuisance regressors.
Effect(s) tested	GLM analyses of whole-brain and brainstem data: we contrasted brain activity during our two task conditions (up- vs. downregulation of pupil size).<br>As complementary analyses, we performed additional GLMs for both whole-brain and brainstem data using pupil diameter recorded throughout the session as regressor of interest in the models.<br>Our primary ROI analyses (performed on brainstem data) was implemented to compare brain activity in up- vs downregulation phases of pupil self-regulation as well as the correlation with recorded pupil diameter throughout the pupil

self-regulation task in our a-priori defined ROIs. Furthermore, we compared up- vs. downregulation phases of self-regulation in additional control ROIs.

Specify type of analysis: ☐ Whole brain ☐ ROI-based ☒ Both

Anatomical location(s): Anatomical locations to test our primary hypothesis were based on a-priori hypothesis and we used probabilistic atlases provided by previous studies

Statistic type for inference (See Eklund et al. 2016)	for the two GLMs (whole-brain data), we used a cluster-wise approach with a threshold of $z > 3.1$ and a cluster p threshold of < 0.05<br>for the GLMs of the brainstem data, we implemented a cluster-wise approach with a threshold of $z > 2.3$ and a cluster p threshold of < 0.05. Additionally, and only reported in the supplementary information, we performed analyses at an uncorrected voxel p threshold of < 0.05.
Correction	Primary ROI analyses contrasting brain activity between up- and downregulation phases as well as the correlation with recorded pupil diameter throughout the task in our a-priori defined ROIs: we used the sequential Bonferroni procedure to correct for multiple comparisons.<br>GLMs for whole-brain data: FWE-corrected for multiple comparisons.<br>GLMs for brainstem data: FWE-corrected for multiple comparisons; additional supplementary analyses were performed at an uncorrected $p < 0.05$.

Models & analysis

n/a	Involved in the study
☒	☐ Functional and/or effective connectivity
☒	☐ Graph analysis
☒	☐ Multivariate modeling or predictive analysis
